# Management of iron deficiency anemia during pregnancy: a midwife-led continuity of care model

**DOI:** 10.3389/fnut.2024.1400174

**Published:** 2024-05-24

**Authors:** Sabahat Naz, Shahira Shahid, Sahir Noorani, Ishrat Fatima, Ali Jaffar, Muhammad Kashif, Nida Yazdani, Uzma Khan, Arjumand Rizvi, Muhammad Imran Nisar, Fyezah Jehan, Zahra Hoodbhoy

**Affiliations:** ^1^Department of Pediatrics and Child Health, The Aga Khan University, Karachi, Pakistan; ^2^The VITAL Pakistan Trust, Karachi, Pakistan; ^3^Provost Office COE, The Aga Khan University, Karachi, Pakistan

**Keywords:** anemia, pregnant women, intravenous iron therapy, midwife-led model of care, primary healthcare (PHC)

## Abstract

**Background:**

Globally, 36.5% of pregnancies are affected by anemia, particularly in low-and middle-income countries, posing significant risks to maternal and perinatal health. In rural Pakistan, 44.3% of pregnant women suffer from iron deficiency, contributing to the high prevalence of anemia. Limited accessibility to antenatal care exacerbates the challenge, necessitating innovative solutions. This study assessed a midwife-led continuity of care model, utilizing intravenous (IV) iron therapy for the management of anemia in Karachi, Pakistan.

**Methods:**

We performed a retrospective analysis of data from a prospective cohort study conducted in two primary healthcare facilities, which employed a community midwife (CMW)-led continuity of care model for antenatal care, including IV iron therapy. We extracted data from February 2021 to March 2022 for women who were diagnosed with anemia based on hemoglobin (Hb) levels, categorized as mild (10.0 to 10.9 g/dL), moderate (7.0 to 9.9 g/dL), or severe (less than 7.0 g/dL). Assessment occurred at the initial antenatal care (ANC) visit to establish baseline anemia severity, and approximately 2 weeks after intravenous (IV) iron therapy administration to evaluate post-treatment changes were considered.

**Results:**

We enrolled 114 pregnant women, where the majority presented with moderate (88.6%) anemia. After IV iron treatment, 48.5% improved to normal-mild levels, while 50% remained unchanged. Severe anemia affected 10.5% at baseline; 42% shifted to moderate and 50% to normal-mild post-treatment, with one remaining unchanged (*p* < 0.001). Among women enrolled in the first and second trimesters, severe anemia improved to normal-mild (50%) and moderate levels (50%) (pre-treatment: *n* = 10, post-treatment: *n* = 0), and moderate anemia decreased by 48% (pre-treatment: *n* = 92, post-treatment: *n* = 47).

**Conclusion:**

Our midwife-led model of care demonstrated an improvement in iron levels among pregnant women. The model addressed the challenges of anemia prevalence in Pakistan and underscored the significance of empowering front-line healthcare providers, such as community midwives (CMWs) for managing these common conditions.

## 1 Introduction

According to the World Health Organization, approximately 36.5% of pregnant women are anemic worldwide; the majority of these (56%) women are from low-and middle-income countries (LMICs) (1). According to the National Nutrition survey, iron deficiency is present in 44.3% of pregnant women in rural Pakistan (2). The World Health Organization (WHO) currently recommends the definition of severe, moderate, and mild anemia in pregnancy based on hemoglobin (Hb) concentrations: less than 7.0 g/dL, 7.0 to 9.9 g/dL, and 10.0 to 10.9 g/dL, respectively (3). There is well-established literature linking adverse maternal and perinatal outcomes with maternal Hb levels lower than 11.0 g/dL. The effects on maternal health include fatigue, compromised immune function, an elevated risk of cardiac diseases and an increased risk of preterm birth, low birth weight, intrauterine deaths (IUFD), low APGAR scores at 5 min, and intrauterine growth restriction (IUGR). Preterm birth and low birth weight are significant contributors to neonatal deaths in Pakistan, which has one of the highest Neonatal Mortality Rates (NMR) globally at 39.4 deaths per 1,000 live births (4). Thus, it is essential to treat anemia in pregnant women (5). Oral iron supplementation is widely prescribed as the first-line treatment for IDA. However, a high frequency of gastrointestinal side effects and the long duration of therapy (4–6 weeks) negatively affect compliance with this therapy (6–8).

In contrast, Intravenous (IV) iron preparations can be safely used for the treatment of IDA during pregnancy and the postpartum period and are more beneficial than oral iron preparations in patients who do not respond to oral iron therapy, have adverse reactions, and have severe anemia requiring rapid iron repletion (9). Administration of IV iron is associated with mild side effects such as rash in 25% of patients, most of which are self-limiting, and about 2% may experience severe allergic reactions. Most of these reactions occurred immediately during the infusion of the test dose (10). IV iron therapy is usually administered in a hospital setting under trained medical staff to oversee these side effects and provide timely treatment to avoid complications (11). This may be difficult to achieve in a low-resource setting such as Pakistan, where women have limited access to antenatal care (ANC); only 41% of the women receive more than one visit, and around 19% of women do not receive any antenatal care (12). In 2006, the Government of Pakistan introduced a new cadre of Community Midwives (CMWs) to address the shortage of skilled health workers. Rural women with more than 10 years of education, typically equivalent to completing secondary education or higher, were recruited and underwent 18 months of midwifery training. They were then deployed back to their towns to provide maternity care. Since then, midwife-led models of care have effectively improved maternal and neonatal outcomes. CMWs now serve as the front-line healthcare workers in various primary healthcare centers (PHCs) across Pakistan to provide ANC, including managing iron deficiency anemia (13).

In this study, we aim to describe the performance of a midwife-led model of care in the management of anemia utilizing IV iron therapy, among pregnant women at a community-based primary healthcare center in Karachi, Pakistan.

## 2 Methodology

### 2.1 Study design and participants

We did a retrospective analysis of data from a prospective cohort study (14) to describe our experience of midwife-led IV iron administration during pregnancy from February 2021 to March 2022 at two primary health care facilities (PHCs) located in peri-urban fishing communities, Rehri Goth and Ibrahim Hyderi in Karachi, Pakistan. These PHCs offer midwifery-led ANC along with ultrasound and laboratory investigations to the community. We extracted data for women with a singleton pregnancy, who presented to the PHCs with anemia and sought ANC services, had Hb levels at their first and last ANC visits, and maternal and perinatal outcomes available. Pregnant women with pre-existing medical conditions such as kidney and liver disease, acute infections, women with severe allergic reactions to iron preparations prior to enrollment were excluded. All women were referred to a secondary-level health facility for labor and birth.

### 2.2 Midwife-led model of care at the PHCs

The community midwives (CMWs) employed at the PHCs have a diploma in midwifery. Their training incorporates didactic and practical components, conducted at allied hospitals and primary health care centers with a focus on continuity of care principles. The midwives at the PHCs underwent regular training sessions and refresher courses to enhance their technical skills. Each center has four midwives providing ANC services to 20–25 pregnant women per day. Thus, each CMW treated 5–6 pregnant women per day. All the CMWs were required to work full time, and one of them served as a supervisor, overseeing the care provided and providing guidance to the CMWs. Throughout the pregnancy, the midwives conducted individual consultations, delivered antenatal care, identified potential complications, and referred for specialist care when necessary. The CMWs followed up with women having health risks at the PHC while collaborating with specialist care. They served as educators, fostering open discussions on birth, breastfeeding, family planning, and reproductive health. All women were provided with the midwives’ phone numbers for emergencies.

**Figure 1:**
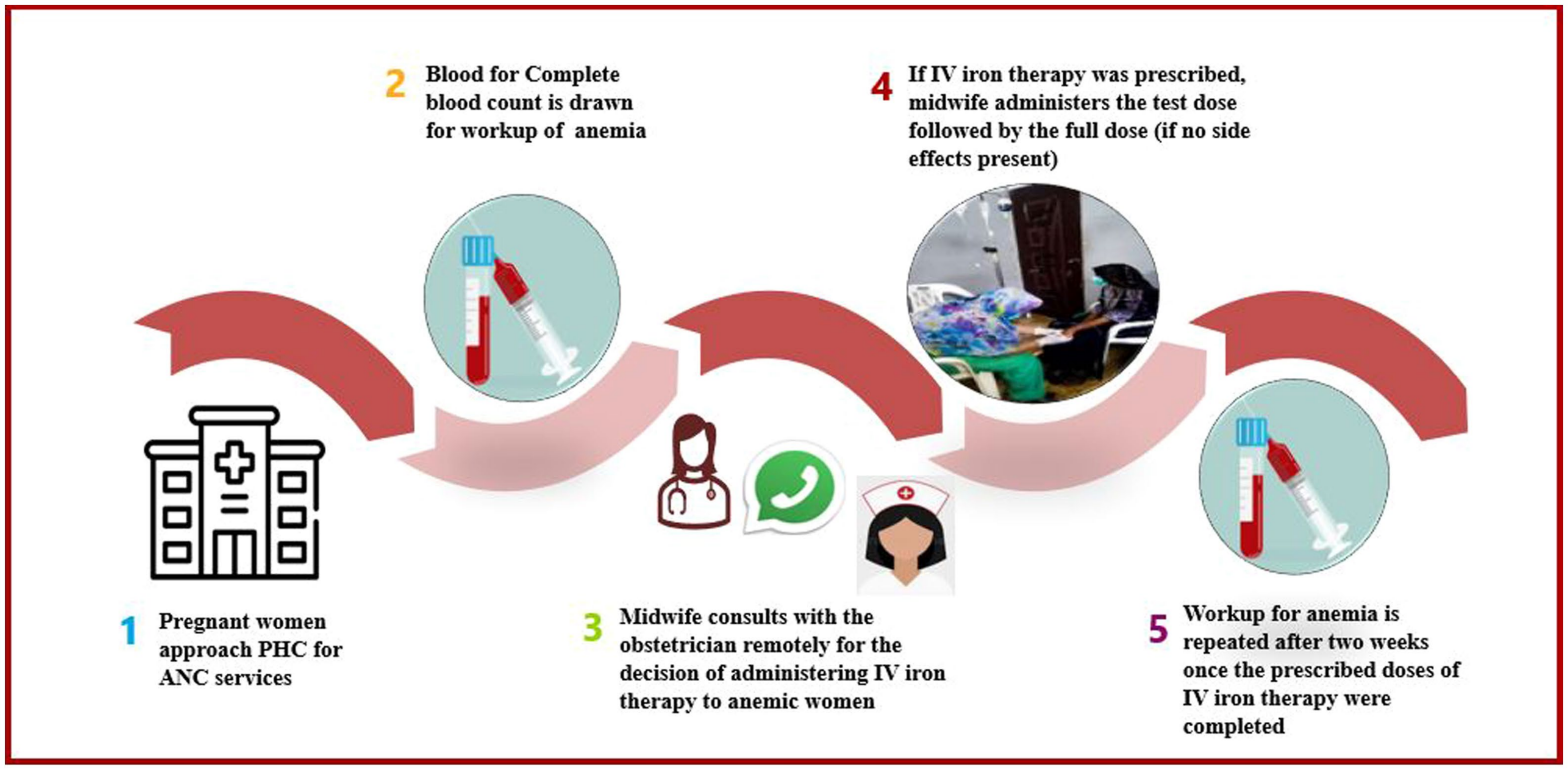
Management of anemia using IV iron therapy at a PHC facility. PHC, Primary Healthcare; ANC, Antenatal Care; IV, Intravenous Iron.

All pregnant women underwent a routine complete blood count (CBC) to assess their anemia status during their first antenatal care visit (3). If a pregnant woman was identified as anemic according to the WHO classification, CMWs consulted the obstetrician at the secondary-level health facility over the phone to discuss further management. If IV iron therapy was prescribed by the obstetrician, a Venofer^®^ (iron sucrose) injection was administered by the CMWs. The dose of iron sucrose administered was calculated using the following formula: the weight of the pregnant women (kg) * [(targeted Hb of 11 – current Hb) * (required increase in Hb)] (15). The total cumulative dose of Venofer^®^ was administered as a maximum of 200 mg not more than 3 times per week; it was ensured that the doses were administered at least 24 h apart. We used a drug-to-diluent concentration of 100 mg Venofer^®^ in 100 mL of 0.9% sodium chloride. The first infusion of Venofer^®^ included a test dose which was administered as 10 mg in 10 mL over 15 min (volume to be infused (VTBI) was 10 mL; IV pump set at 100 mL/h), and if no side effects were reported, the remaining dose of 90 mg in 90 mL infusion was given over 40–45 min at an infusion rate of not more than 200 mL/h. Subsequent doses of 100 mg in 100 mL of 0.9% sodium chloride were administered at a maximum pump rate of 200 mL/h under the supervision of the midwife, and Hb levels were repeated 2 weeks after the prescribed dose was administered (Figure 1).

As part of the midwife-led model of antenatal care, we incorporated other measures to improve anemia, particularly focusing on dietary counseling during every ANC visit along with folic acid supplementation. However, we ensured that women were not concomitantly taking oral supplements with IV therapy. Women who were found resistant to treatment were referred to a secondary-level facility for further evaluation and management to mitigate potential complications associated with anemia.

Data regarding the women’s ANC visits and postnatal home visits were electronically captured from the PHC registries through a custom-built Android application by community health workers and stored on a PostgreSQL database. The data was cross-checked with the registries to ensure validity and that all eligible births were included. All identifications were removed before the data were transferred to the final database at Aga Khan University servers.

### 2.3 Training for administration of IV iron

The community midwives were trained based on appropriate management protocols, including regular refreshers covering the safe administration of IV iron therapy. The training for IV iron was over 2 days, with weekly refreshers of 1 h every quarter. They were supervised by the midwifery school staff and obstetricians for administration of IV iron. The assessment was based on a checklist to ensure that the midwife asked about any possible allergic reactions, administered the test dose correctly, followed by administration of the full dose and monitoring of vitals. CMWs were trained to promptly identify signs of an adverse event during IV iron administration and to initiate immediate interventions depending on the severity of the reaction.

Mild reactions, characterized by symptoms such as hypertension, slight chest tightness, or urticaria, prompted a 15-min pause in the infusion, during which the patient’s response was assessed, and the obstetrician was contacted. If improvement was noted, the infusion was cautiously resumed at a reduced rate with continued observation for at least 1 h. Moderate hypersensitivity reactions, characterized by dyspnea, hypotension, chest tightness, and tachycardia, necessitated immediate cessation of the infusion, whereas severe or life-threatening hypersensitivity reactions (anaphylaxis), marked by respiratory distress required emergency referral after administering the first dose of dexamethasone.

We obtained written informed consent from all pregnant women prior to IV administration. The informed consent process included explaining to women the potential risks and benefits associated with IV iron therapy. By obtaining consent, we ensured that women were fully aware of the procedure and its implications, thereby promoting patient autonomy. Only CMWs trained comprehensively in IV administration and management of adverse events were allowed to administer the doses. Standardized procedures were followed for the calculation of the iron doses, monitoring of vital signs and clinical symptoms during and after infusion, and management of adverse events.

### 2.4 Statistical analysis

Data related to sociodemographic, clinical, and birth-related characteristics were presented as mean ± SD or frequencies and percentages as appropriate. Anemia categories were defined as severe, moderate, and mild based on hemoglobin (Hb) concentrations: less than 7.0 g/dL, 7.0 to 9.9 g/dL, and 10.0 to 10.9 g/dL, respectively. We compared changes in the severity of anemia pre-and post-treatment with IV iron using a McNemar test. A *p*-value <0.05 was considered significant. We also presented frequencies and percentages for place of birth, mode of birth, primary birth attendant, gestational age at delivery, birth weight, and neonatal death based on IV iron dose received by the women. Data were analyzed using Stata^®^ (Version 14.2 Statacorp Texas, United States).

### 2.5 Ethics

This study was approved by the Ethical Review Committee of Vital Pakistan Trust (Reference number: 001-VPT-IRB-20).

## 3 Results

A total of 114 pregnant women were enrolled in the study. Table 1 describes the sociodemographic characteristics of the study participants. We enrolled 35.1% (n = 40) of the women in the first trimester, 55.3% (*n* = 63) women enrolled in the second trimester, and 9.6% (*n* = 11) women in their third trimester. The mean age of the pregnant women was 26.17 +/− 5.37 years. Almost 66% (*n* = 75) of the women received no formal education, and 21.1% (*n* = 24) attended school up to secondary level. Approximately 34.2% (*n* = 39) of the pregnant women used gutka/tobacco, 18.4% (*n* = 21) were severely malnourished (<21), and 18.4% (*n* = 21) were moderately malnourished (21 to <23). During the study period, 43.9% (*n* = 50) of the pregnant women received a single dose, 32.5% (*n* = 37) received two doses, and 23.7% (*n* = 27) received three or more doses of IV iron.

**Table 1:** Clinical characteristics of the study participants.

Characteristics	*N* = 114 mean ± SD
Age in years	26.17 ± 5.37
BMI (kg/m^2^)	21.69 ± 0.37
Gestational age at enrollment	24.8 ± 4.9

Characteristics	*n* (%)
*Gestational age (trimester-wise) at enrollment*	
First trimester Second trimester Third trimester	40 (35.1) 63 (55.3) 11 (9.7)
*Education*	
No formal education Primary Secondary Higher	75 (65.8) 12 (10.5) 24 (21.1) 3 (2.6)
*Employment status*	
Unemployed Skilled manual work Unskilled manual work	68 (60.7) 27 (24.1) 17 (15.2)
*ANC visits*	
≤4	21 (18.4)
5 to 8	70 (61.4)
>8	23 (20.2)
*Presence of one or more risk factors*^a^ Hypertension Tuberculosis History of blood transfusion Previous cesarean section	8 (7.0) 3 (2.6) 1 (0.9) 1 (0.9) 5 (4.4)
*Consumed gutka or tobacco*	39 (34.2)
*MUAC* (cm)	
Severely malnourished (<21) Moderately malnourished (21 to <23) Normal (> = 23)	21 (18.4) 21 (18.4) 72 (63.2)
*Anemia status (g/dL)*	
Mild (10–10.9)	1 (0.9)
Moderate (7–9.9)	101 (88.6)
Severe (<7)	12 (10.5)
*Venofer dose*	
1 Dose	50 (43.9)
2 Doses	37 (32.5)
≥3 Doses	27 (23.7)

a Self reported.

ANC, Antenatal care services; MUAC, Mid-upper arm circumference; BMI, Body mass index.

The majority of pregnant women had moderate anemia (88.6%, *n* = 101) at baseline. Of these, 48.5% (*n* = 49) converted to normal-mild after correction with IV iron, whereas 50.5% (*n* = 51) remained unchanged. We observed 10.5% of the women to have severe anemia (*n* = 12) at baseline, 42% (*n* = 5) shifted to moderate, and 50% (*n* = 6/12) to normal to mild anemia after receiving treatment, whereas 1 remained unchanged (*p* = <0.001) (Table 2).

**Table 2:** Distribution of anemia status before and after treatment with IV iron.

Anemia status		After treatment *n* (%)			
		^a^Normal to mild	Moderate	Severe	Total
Before treatment *n* (%)	Mild	1 (100)	0	0	1 (0.9)
	Moderate	49 (48.5)	51 (50.5)	1 (1)	101 (88.6)
	Severe	6 (50)	5 (42)	1 (8)	12 (10.5)
	Total	56 (49)	56 (49)	2 (2)	114
	McNemar test	*p* = <0.001			

a This category included women with normal and mild anemia after receiving treatment.

When comparing the change in anemia status during the first and second trimesters, severe anemia improved to normal-mild (50%) and moderate levels (50%) (pre-treatment: *n* = 10, post-treatment: *n* = 0), whereas there was a 48% reduction in moderate anemia (pre-treatment: *n* = 92, post-treatment: *n* = 47) (Table 3). However, among women enrolled in the third trimester, there was a 55% reduction in moderate anemia (pre-treatment: *n* = 9, post-treatment: *n* = 4) (Table 4). In our study, 14% (*n* = 16) of the women reported minor side effects, including dizziness (15%, *n* = 8), redness/swelling of arms or legs (19%, *n* = 3), nausea (12.5%, *n* = 2), irregular fetal heart rate (6%, *n* = 1), headache (6%, *n* = 1), and itching (6%, *n* = 1).

**Table 3:** Distribution of anemia severity before and two weeks after treatment among women enrolled in the first and second trimesters, n (%).

Anemia status		After treatment			
		^a^Normal to mild	Moderate	Severe	Total
Before treatment	Mild	1 (100)	0	0	1 (1.0)
	Moderate	44 (48)	47 (51)	1 (1.0)	92 (89.3)
	Severe	5 (50)	5 (50)	0	10 (9.7)
	Total	50 (49)	52 (50)	1 (1.0)	103

a This category included women with normal and mild anemia after receiving treatment.

**Table 4:** Distribution of anemia severity before and 2 weeks after treatment among women enrolled in the third trimester, *n* (%).

Anemia status		After treatment			
		^a^Normal to mild	Moderate	Severe	Total
Before treatment	Mild	0	0	0	0
	Moderate	5 (55)	4 (45)	0	9 (81.8)
	Severe	1 (50)	0	1 (50)	2 (18.2)
	Total	6 (55)	4 (36)	1 (9)	11

a This category included women with normal and mild anemia after receiving treatment.

The pregnancy-related outcomes of the study participants are described in Table 5. The mean gestational age at birth was 38.56 ± 1.70 weeks. Preterm deliveries accounted for 10.5% (*n* = 12) of the live births. The majority of deliveries took place in a facility-based setting n = 97 (85.1%), with 14.9% percent occurring at home. Spontaneous vaginal birth was the predominant mode of delivery, constituting 81.6% (*n* = 93), while cesarean sections accounted for 18.4% (*n* = 21). The primary birth attendants were skilled attendants in the majority of the deliveries (85.1%, *n* = 97). There were (98.3%, *n* = 112) livebirths and (1.8%, *n* = 2) stillbirths. The average birth weight was 2.9 ± 0.86 kg. There was one early neonatal death reported (0.9%) and no late neonatal deaths in the study population. There was no preterm birth among women who received 3 or more IV iron doses. Stillbirths accounted for 0.9% in single and two-dose groups, and no stillbirths were reported in the three or more-dose group (Table 5).

**Table 5:** Pregnancy-related outcomes among the study participants.

Pregnancy related outcomes	1 Dose (*n* = 50) mean ± SD	2 Doses (*n* = 37) mean ± SD	≥ 3 Doses (*n* = 27) mean ± SD	Total (*N* = 114) mean ± SD
GA at birth (weeks)	38.27 ± 1.58	38.57 ± 2.09	39.09 ± 1.17	38.56 ± 1.70
Birth weight (kg)	3.02 ± 1.22	2.88 ± 0.36	2.81 ± 0.51	2.9 ± 0.86

Pregnancy related outcomes	*n* (%)	*n* (%)	*n* (%)	*n* (%)
Preterm birth (<37 weeks GA)	8 (7.0)	4 (3.5)	0	12 (10.5)
Place of birth				
Home	7 (6.1)	8 (7.0)	2 (1.8)	17 (14.9)
Facility based	43 (37.7)	29 (25.4)	25 (22.0)	97 (85.1)
Mode of birth				
SVD	36 (31.6)	33 (29)	24 (21)	93 (81.6)
Cesarean	14 (12.3)	4 (3.5)	3 (2.6)	21 (18.4)
Primary birth attendant				
Skilled	43 (37.7)	29 (25.4)	25 (21.9)	97 (85.1)
Unskilled	7 (6.1)	8 (7.0)	2 (1.8)	17 (14.9)
Pregnancy outcome				
Live Birth	49 (43)	36 (31.6)	27 (23.7)	112 (98.3)
Stillbirth	1 (0.9)	1 (0.9)	0	2 (1.8)
Neonatal deaths	0	1 (0.9)	0	1 (0.9)

GA, Gestational Age; SD, Standard Deviation; SVD, Spontaneous Vaginal Birth.

## 4 Discussion

Iron deficiency anemia is one of the most common medical complications of pregnancy. Our midwife-led model of care for managing anemia through IV iron therapy demonstrated a 90% reduction in the prevalence of severe and approximately 50% reduction in the prevalence of moderate anemia throughout the trimesters at the PHCs without a need for referral to a secondary health facility. In addition, our study also demonstrated minimal side effects, with dizziness, redness/swelling of arms or legs, and nausea being the three commonly reported side effects.

Midwives are an important cadre that can take on various tasks related to maternal and reproductive health. To help alleviate the burden on the healthcare system, in 2006, the Government of Pakistan initiated the community midwifery program to improve access to maternity services across Pakistan (16). Previous evidence has shown that midwives served a key role in the community by providing services such as family planning, cervical screening, and treatment of sexually transmitted infections (17–19). However, these models, specifically those from Pakistan, seem more focused on clinical knowledge than technical and soft skills (17, 18). Although not formally tested, our study suggests that the midwives could efficiently administer injections with appropriate training, highlighting their capacity to integrate technical skills with the necessary clinical knowledge.

Our findings align with previous literature where a higher proportion of pregnant women with severe and moderate anemia achieved mild (10.0 to 10.9 g/dL) to normal (≥ 11 g/dL) status of Hb levels after receiving IV iron therapy (20, 21). However, these studies were conducted in healthcare settings where medical officers administered IV iron therapy instead of midwives (20, 21). In contrast, our study demonstrated that using midwives under the telephonic supervision of an obstetrician can be equally competent for managing IDA in community settings where access to higher-level healthcare facilities is challenging in terms of availability, time, and cost.

Our midwife-led model of care for managing anemia through IV iron therapy observed minor adverse reactions among 14% of pregnant women, such as dizziness, redness/swelling of arms or legs, and nausea, and none of them required discontinuation of the therapy. Our study findings are aligned well with the findings of Kriplani et al., which also found minor side effects among pregnant women (14%) who received IV iron therapy, including nausea, vomiting, diarrhea, and fever (21). However, this study administered IV iron therapy in a well-equipped healthcare setting.

Despite the effective role of IV iron therapy in treating IDA among pregnant women, the burden of anemia remains persistently high, especially in rural and peri-urban communities (20, 22). Several challenges exist to the implementation of IV iron therapy. The administration of IV iron is usually available in secondary care hospitals under close monitoring of registered nursing staff consulting with assigned physicians. Women residing in low-resource communities have limited income sources with poor access to health facilities, making them less likely to benefit from IV iron therapy (6). Another reason is the fear of systemic side effects associated with IV administration (13). Our study overcame these barriers through the availability of trained midwives, enabling pregnant women to undergo treatment at a primary-level facility without needing referrals.

The collaboration between midwives and obstetricians ensured comprehensive care, while counseling regarding IV iron therapy promoted acceptance and adherence. In our study, we found some facilitators and barriers to the implementation of midwife-administered IV iron therapy during our regular monthly interactions with the midwives. They mentioned that women appreciated that they could get treatment near their homes, and this saved time and cost for traveling to a distant facility. In contrast, the barriers included the absence of equipment such as a crash cart. However, we observed minor adverse reactions among pregnant women, and none of them required discontinuation of therapy. In addition, the midwives mentioned that even though they have never seen a woman have a severe allergic reaction, they are worried about that situation and remain very alert while administering this.

The pregnant women attending the PHCs were young, the majority had received no formal education, and there was a high prevalence of tobacco use and malnutrition, highlighting the multifaceted challenges faced by pregnant women in the community. However, their pregnancy-related outcomes depicted a favorable scenario, with the majority of deliveries occurring in a facility-based setting and a high proportion of spontaneous vaginal deliveries in the presence of skilled birth attendants. The low stillbirth rate and neonatal death rate further support the possible positive impact of IV iron administration and midwife-led care, which can be explored in further research. The findings of the study require cautious interpretation since various factors beyond IV iron administration, such as socioeconomic status, food insecurity, and previous pregnancy history, may influence the observed effects of IV iron therapy on anemia and maternal and perinatal health.

Our study has certain strengths and limitations. To the best of our knowledge, this study is one of the first to assess the management of midwife-led IV iron therapy among pregnant women in a primary healthcare center, which is a novel approach for addressing a prevalent medical complication and improving accessibility to healthcare in underserved areas. Since we performed a retrospective analysis of data from a prospective cohort study (14) for women who received IV iron therapy for managing anemia, the absence of a comparison group is an important limitation of the study. In addition, we relied solely on hemoglobin levels for diagnosing anemia due to the lack of resources for conducting further investigation. However, as indicated by the National Nutrition Survey, approximately 60% of anemia in pregnancy is attributed to iron deficiency alone (2), thus leading to this treatment option.

## 5 Conclusion

The midwife-led model of care managing anemia among pregnant women in a primary healthcare (PHC) setting resulted in an improvement in their iron levels. This model can be implemented in remote settings, ensuring a safe and accessible approach to managing one of the most prevalent conditions impacting pregnant women.

## Data availability statement

The raw data supporting the conclusions of this article will be made available by the authors, without undue reservation.

## Ethics statement

The studies involving humans were approved by VITAL Pakistan Trust, Karachi, Pakistan. The studies were conducted in accordance with the local legislation and institutional requirements. The participants provided their written informed consent to participate in this study.

## Author contributions

SNa: Formal analysis, Investigation, Methodology, Project administration, Resources, Validation, Visualization, Writing – original draft, Writing – review & editing. SS: Formal analysis, Methodology, Validation, Visualization, Writing – original draft, Writing – review & editing. SNo: Formal analysis, Methodology, Validation, Visualization, Writing – original draft. IF: Investigation, Methodology, Project administration, Resources, Validation, Writing – original draft. AJ: Formal analysis, Investigation, Methodology, Project administration, Resources, Validation, Writing – original draft. MK: Data curation, Formal analysis, Investigation, Software, Validation, Visualization, Writing – original draft. NY: Investigation, Methodology, Project administration, Resources, Validation, Writing – original draft. UK: Formal analysis, Investigation, Methodology, Project administration, Resources, Validation, Writing – original draft. AR: Data curation, Formal analysis, Investigation, Software, Validation, Visualization, Writing – original draft. MN: Conceptualization, Formal analysis, Funding acquisition, Investigation, Methodology, Project administration, Resources, Software, Supervision, Validation, Visualization, Writing – original draft, Writing – review & editing. FJ: Investigation, Methodology, Project administration, Resources, Software, Supervision, Validation, Visualization, Writing – original draft, Writing – review & editing, Conceptualization, Data curation, Formal analysis, Funding acquisition. ZH: Conceptualization, Data curation, Formal analysis, Funding acquisition, Investigation, Methodology, Project administration, Resources, Software, Supervision, Validation, Visualization, Writing – original draft, Writing – review & editing.
